# Isolation and Characterization of Six *AP2/ERF* Transcription Factor Genes in *Chrysanthemum nankingense*

**DOI:** 10.3390/ijms16012052

**Published:** 2015-01-19

**Authors:** Chunyan Gao, Peiling Li, Aiping Song, Haibin Wang, Yinjie Wang, Liping Ren, Xiangyu Qi, Fadi Chen, Jiafu Jiang, Sumei Chen

**Affiliations:** 1 College of Horticulture, Nanjing Agricultural University, Nanjing 210095, China; E-Mails: 2011104110@njau.edu.cn (C.G.); 2011204026@njau.edu.cn (P.L.); aiping_song@aliyun.com (A.S.); hb@njau.edu.cn (H.W.); 2011104112@njau.edu.cn (Y.W.); 2012204032@njau.edu.cn (L.R.); 2012204028@njau.edu.cn (X.Q.); chenfd@njau.edu.cn (F.C.); jiangjiafu@njau.edu.cn (J.J.); 2 Jiangsu Province Engineering Lab for Modern Facility Agriculture Technology & Equipment, Nanjing 210095, China

**Keywords:** hormone, PCR, stress response, transcription pattern

## Abstract

The AP2/ERF family of plant transcription factors (TFs) regulate a variety of developmental and physiological processes. Here, we report the isolation of six *AP2/ERF* TF family genes from *Chrysanthemum nankingense.* On the basis of sequence similarity, one of these belonged to the Ethylene Responsive Factor (ERF) subfamily and the other five to the Dehydration Responsive Element Binding protein (DREB) subfamily. A transient expression experiment showed that all six AP2/ERF proteins localized to the nucleus*.* A yeast-one hybrid assay demonstrated that *CnDREB1-1*, *1-2* and *1-3* all function as transactivators, while *CnERF1*, *CnDREB3-1* and *3-2* have no transcriptional activation ability. The transcription response of the six TFs in response to wounding, salinity and low temperature stress and treatment with abscisic acid (ABA), salicylic acid (SA) and jasmonic acid (JA) showed that *CnERF1* was up-regulated by wounding and low temperature stress but suppressed by salinity stress. The transcription of *CnDREB1-1*,* 1-2* and* 1-3* was down-regulated by ABA and JA to varying degrees. *CnDREB3-1* and *3-2* was moderately increased or decreased by wounding and SA treatment, suppressed by salinity stress and JA treatment, and enhanced by low temperature stress and ABA treatment.

## 1. Introduction

Plants have evolved a diversity of responses to external stress. Many of these rely on signalling provided by the phytohormones abscisic acid (ABA), jasmonate (JA), salicylic acid (SA) or ethylene [1]. A large class of rapid defense responses are mediated by transcription factors (TFs) [2]. The AP2/ERF TFs belong to a particularly diverse family of TFs which are recognized by the presence of a characteristic 57–66 residue long AP2/ERF DNA binding domain [3]. The AP2/ERF family is divided into four major sub-families based on sequence similarity and the number of AP2/ERF domains present; these sub-families are AP2, DREB/ C-repeat binding factor (CBF), ERF and RELATED TO ABI3/VP1 (RAV) [4,5]. DREB and ERF TFs have frequently been implicated in the response to drought, salinity, rapid changes in temperature and disease [6]. AP2 TFs harbor two AP2/ERF domains, and many of them participate in the regulation of development [7]. The RAV subfamily TFs contain one AP2/ERF domain and one B3 domain, interact with either ethylene or brassinosteroids [8], and form part of the response to biotic and abiotic stress [9]. The DREB/CBF and ERF TFs each harbor only one AP2/ERF domain. DREB/CBF TFs are an important component of the plant abiotic stress tolerance [10], while the ERF TFs participate in the response to both biotic and abiotic stress [11]. The *Arabidopsis thaliana* genome harbors 147 AP2/ERF TFs [12], rice 167 [13], poplar 200 [5], grapevine 132 [4], soybean 148 [14] and bread wheat 117 [15].

The diploid species* Chrysanthemum nankingense* is native to China [16]. It is used both as a vegetable and as a source of anti-cancer flavonoids and aromatic oils. The species also features a number of genes which have proven to be beneficial in chrysanthemum breeding [17]. As yet, the TF content of this species has not been explored. Here, the aim was to isolate and characterize a number of *AP2/ERF* TFs from *C. nankingense.*

## 2. Results and Discussion

### 2.1. Identification of Six AP2/ERF TFs in C. nankingense

**Figure 1:**
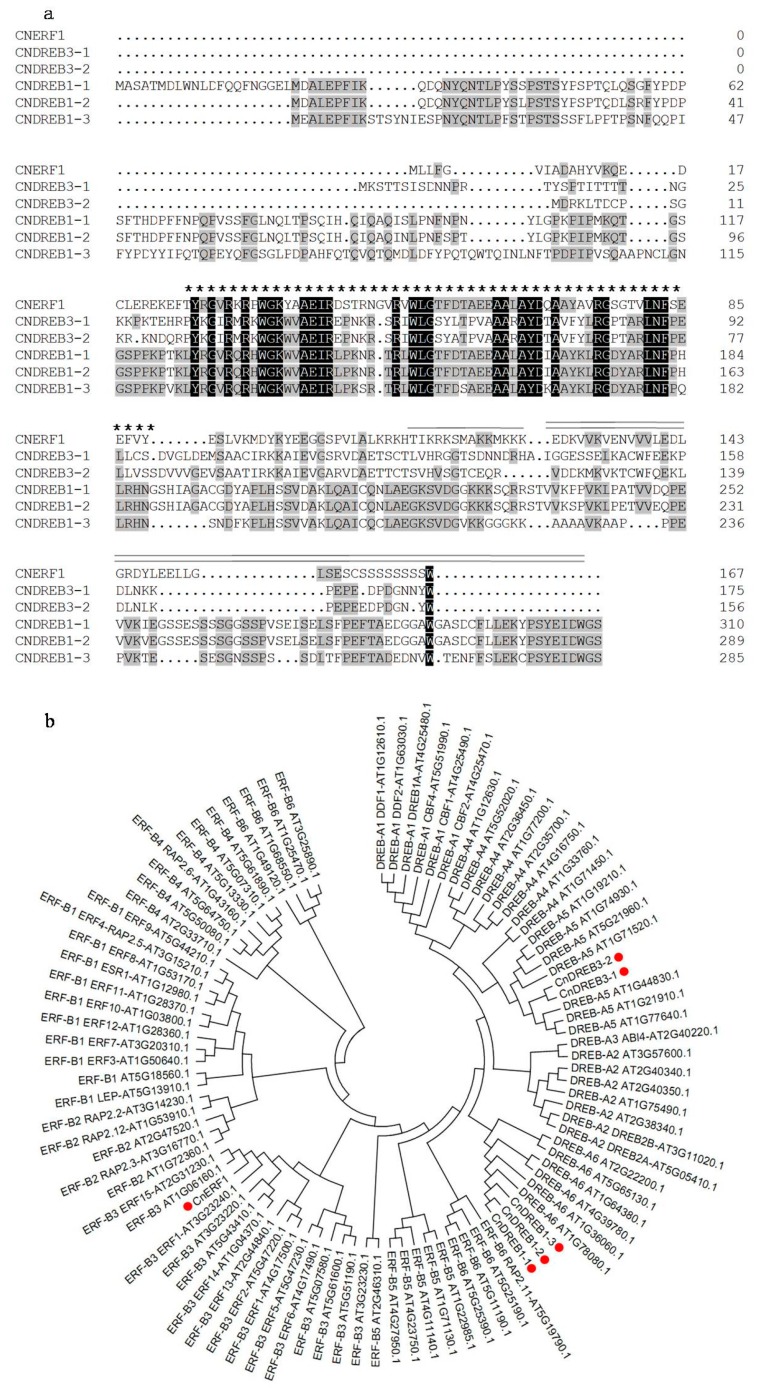
Deduced peptide sequences of the *CnAP2/ERF* transcription factor (TF) products and their phylogenetic relationship with *A. thaliana* homologs. (**a**) The deduced polypeptide sequences; residues shared by at least three of the six sequences are shown shaded, whereas those conserved across all six polypeptides are marked in dark grey. Asterisks indicate the conserved DNA-binding AP2/ERF domain, a double overline indicates the putative acidic domain and a black underline indicates the putative nuclear localization signal; (**b**) Phylogeny of the *CnAP2/ERF* TF products. Dots indicate likely homologs.

Six AP2/ERF full-length cDNAs were isolated and designated *CnERF1*,* CnDREB1-1*,* 1-2*,* 1-3*,* 3-1* and* 3-2*. Their length varied from 683 to 1246 bp, and their predicted translation products comprised from 156 to 311 residues. *CnERF1* belongs to ERF subfamily group B-3, while *CnDREB1-1*,* 1-2* and *1-3* all belong to DREB subfamily A-6 and *CnDREB3-1* and *3-2* to DREB group A-5 (Table 1, Figure 1b). All six enc00oded proteins having a single 64 residue AP2 domain, except for CnERF1 (65 residues) (Figure 1a). Analysis using Smart online software (available online: http://smart.embl-heidelberg.de/) showed that the conserved residues in the *CnERF1* AP2/ERF were A14 and D19. The corresponding positions in *CnDREB1-1*, *1-2* and *1-3* were occupied by, respectively valine and leucine, whereas in *CnDREB3-1* and *3-2*, the 19th residue was glutamic acid. The 14th and 19th residues are both located on the β-sheet of the AP2/ERF domain.

**Table 1:** CnAP2/ERF TF sequences and the identity of likely *A. thaliana* homologs.

Gene	GenBank Accession No.	cDNA Length (bp)	Amino Acids Length (aa)	AtAP2/ERF Orthologs	Locus Name	*E*-Value
*CnERF1*	KF986840	683	167	ERF1B	AT3G23240	1 × 10^−38^
*CnDREB1-1*	KF986841	1241	311	RAP2.4	AT1G78080.1	2 × 10^−69^
*CnDREB1-2*	KF986842	1164	290	RAP2.4	AT1G78080.1	3 × 10^−69^
*CnDREB1-3*	KF986843	1246	286	ERF055	AT1G78080.1	8 × 10^−42^
*CnDREB3-1*	KF986844	881	175	ERF008	AT2G23340.1	4 × 10^−45^
*CnDREB3-2*	KF986845	658	156	ERF008	AT2G23340.1	1 × 10^−48^

Many examples have been provided where the heterologous expression of a TF can enhance abiotic stress tolerance [18,19]. Five of the six *C. nankingense* TFs isolated here belong to the DREB class, and one to the ERF class; both these types of TF are heavily implicated in the regulation of the stress response [20]. TFs are an attractive target for engineering the stress tolerance of crop plants [21], since a single TF commonly regulates a whole suite of genes, and thus can control a whole adaptive pathway [22].

### 2.2. Subcellular Localization of Cnap2/Erf Products and the Transcription Activation of the TFs

**Figure 2:**
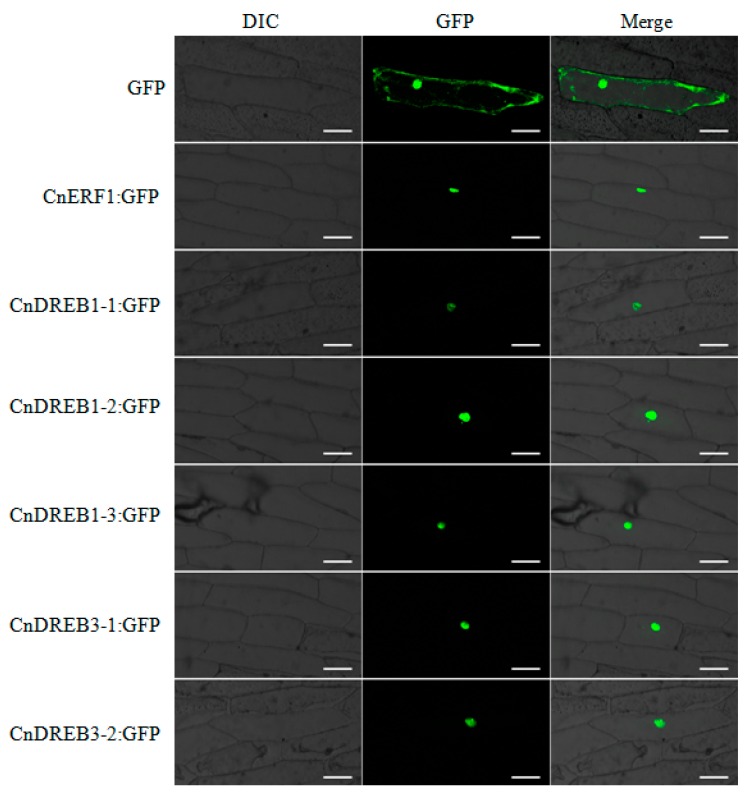
Localization of transiently expressed *CnAP2/ERF* TF products in onion epidermal cells. The upper row shows the control *35S::GFP* signal, and each of the lower rows the signal from one of the *35S::CnAP2/ERF-GFP* transgenes. The left panel shows bright field images, the middle one green fluorescence signals detected at 488 nm and the right one the merged Green Fluorescent Protein (GFP) and bright field images. Bar: 50 μm.

**Figure 3:**
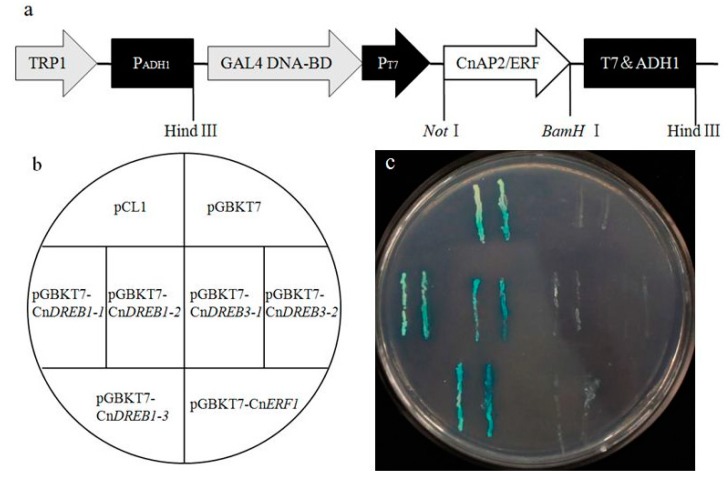
Transcriptional activation activity of the CnAP2/ERF TFs. (**a**) The structure of the *pGBKT7-CnAP2/ERF* plasmid; (**b**) the arrangement of yeast strains on the plate; (**c**) the growth of transformed yeast cells on SD/-His-Ade + 20 mg/mL X-α-gal medium. pCL1 and pGBKT7 are positive and negative controls, respectively.

The outcome of the transient transformation of the six *CnAP2/ERF* TFs is shown in Figure 2. As expected for a TF, the CnAP2/ERF-GFP signal was localized predominantly in the nucleus, while the control GFP only transgene was expressed throughout the cell. Based on the yeast one hybrid assay, C*nDREB1-1*,* 1-2* and *1-3* all showed transcription activation activity in yeast (Figure 3), while *CnERF1*, *CnDREB3-1* and *3-2* did not.

The product of all six of the TFs localized, as would be expected for a TF, in the nucleus (Figure 2), while the test of their transactivation activity was positive for three of them (*CnDREB3-1*,* 3-2* and *CnERF1*, see Figure 3). The EAR (ERF-associated amphiphilic repressor) motif has been proposed to convert transcriptional activators into dominant repressors in plant cells [23], and this motif is present in both *CnDREB3-1* and* 3-2*, inferring that these two TFs might be repressors. A-5 subgroup of AP2/ERF family contains a clade of proteins that have a functional EAR motif at their *C*-terminus [24,25]. Moreover, genes encoding these EAR motif-containing proteins are upregulated in transgenic plants overexpressing *DREB1A/CBF3* or *DREB2A CA* [26,27]. Overexpression of these genes results in reduced expression of *DREB1/CBF* and *DREB2* target genes under cold and dehydration, respectively [28,29]. Taken together, it is possible that *CnDREB3-1* and *3-2* function in negative feedback regulation of the DREB1/CBF and DREB2 pathways [30]. The product of the tomato TF *LeERF1* binds rather weakly to its target GCC box [31], while that of *AtERF1* is quite sensitive to changes in the GCC box sequence (AGCCGCC) [32]. The binding activity of CnERF1 to GCC box remained unknown. In addition, CnERF1 does not possess EAR motif; however, whether the *CnERF1* acts as transcriptional repressor remains to be studied.

### 2.3. Transcription of CnAP2/ERF TFs in Response to Abiotic Stress

**Figure 4:**
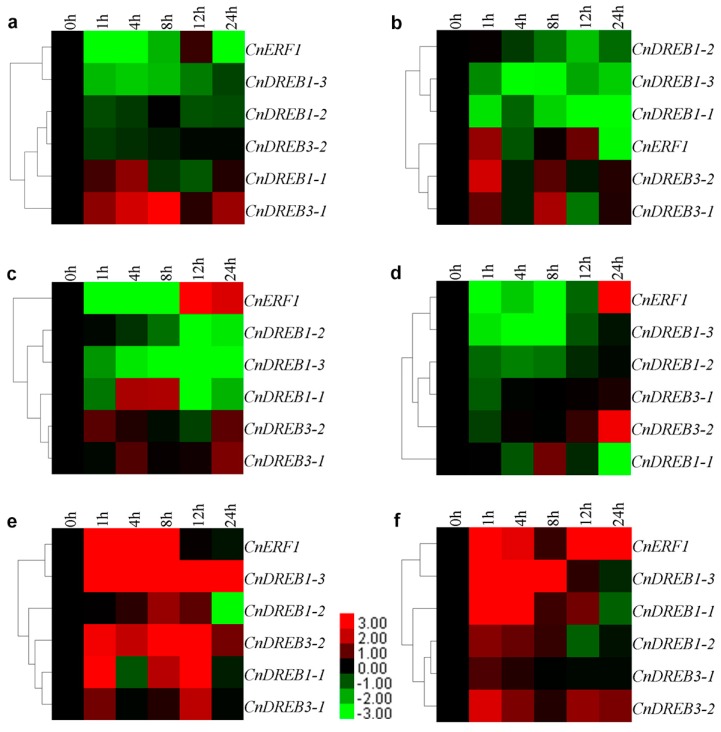
Differential transcript abundance of the *CnAP2/ERF* TFs in response to (**a**) salinity stress; (**b**) abscisic acid (ABA) treatment; (**c**) salicylic acid (SA) treatment; (**d**) jasmonic acid (JA) treatment; (**e**) low temperature stress; and (**f**) wounding. Green cells indicate suppressed and red ones enhanced levels of transcript abundance compared to the relevant control. Black cells represent no significant change of transcript abundance.

Transcript abundance of *CnDREB1-1* was raised by salinity stress, peaking 4 h after the stress was imposed, whereas *CnDREB3-1* was up-regulated throughout the salinity stress episode, peaking at 8 h. The other four TFs were down-regulated to differing degrees; thus *CnDREB1-3* was down-regulated throughout, while the abundance of *CnERF1*,* CnDREB1-2* and* 3-2* transcript recovered to the background level after some time (Figure 4a). *CnDREB3-1* and *3-2* were substantially up-regulated after a 1 and 8 h exposure to ABA, but *CnDREB1-1*,* 1-2* and *1-3* were down-regulated throughout the treatment, and *CnERF1* was only marginally induced at 1 and 12 h (Figure 4b). The effect of SA treatment was to initially induce *CnDREB3-1* and *3-2* transcription, but the transcript abundance then declined, only to increase again by 24 h. *CnDREB1-2* and *1-3* were both strongly down-regulated, while *CnDREB1-1* was induced initially and *CnERF1* at a somewhat later time (Figure 4c). When subjected to JA treatment, the transcription of both *CnDREB3-1* and *3-2* was largely unaffected, that of *CnERF1* was increased at the end of the period and the other three TFs were all repressed to differing extents (Figure 4d). The low temperature stress induced *CnDREB1-3* and *3-2* throughout the treatment, while it caused an oscillation in the transcription of *CnDREB1-1* and *3-1.* *CnERF1* was up-regulated within the first 8 h, but later was suppressed, and *CnDREB1-2* behaved similarly (Figure 4e). Wounding increased the transcript abundance of all six TFs initially, with *CnDREB1-1* and *1-3* peaking at 4 h, *CnERF1* and* CnDREB3-2* still substantially showing up-regulation at 24 h, and *CnDREB1-3*,* 1-1*, *1-2* and* 3-1* being variously suppressed towards the end of the stress episode (Figure 4f). All obtained data were displayed in Table S1.

The differential responses of the AP2/ERF TFs to the various abiotic stresses and hormone treatments suggested that each TF has a specific physiological role. Emerging evidence suggests that pathways regulated by ABA, SA, JA and ethylene are involved in a substantial amount of crosstalk with biotic and abiotic stress pathways [33]. The SA signal transduction pathway can be antagonistic to the ethylene/JA pathway and JA is considered to be a key regulator of stress-induced gene expression across the plant kingdom [34]. The tomato TF *Pti4* and the *A. thaliana* TF *AtERF1* are both induced by SA as well as by JA [32].* AtERF1* plays a positive role in drought, salt and heat stress tolerance by stress-specific gene regulation, which integrates JA and abscisic acid signals [35]. Here, *CnERF1* was up-regulated by wounding and low temperature, but suppressed by salinity and ABA (Figure 4), suggesting its involvement in the response to the former two stresses. In case of DREB genes, members of the DREB1/CBF subfamily are rapidly induced in response to low temperature, and when constitutively expressed, enhance freezing tolerance in *A. thaliana* [36]. Some DREB TFs are regulated by ABA, high-salt and cold [37], as was the case for *CnDREB3-1*,* 3-2* and* 3-3*; the transcription of these same TFs was also affected by JA. CnDREB1-1 and 1-2 was moderately increased/decreased by wounding and SA treatment, suppressed by salinity stress and JA treatment, and enhanced by low temperature stress and ABA treatment; the implication is that both *CnDREB1-1* and *1-2* are involved in the low temperature and the ABA response. Indeed, *CnDREB1-1* and *1-3* were more likely involved in the abiotic stress response than *CnDREB1-2*.

## 3. Experimental Section

### 3.1. Plant Materials and Stress Treatments

The accession of *C. nankingense* used was obtained from the Chrysanthemum Germplasm Resource Preserving Centre, Nanjing Agriculture University, Nanjing, China. The plants were propagated by cutting and grown in a 1:1 (*v*/*v*) mixture of garden soil and vermiculite with no additional fertilizer provided. Rooted seedlings were maintained in a greenhouse. Young plants at the 6–8 leaf stage were used to assess the transcription response to abiotic stress and phytohormne treatment. Salinity stress was imposed by transferring young plants for one day to a liquid medium containing 200 mM NaCl [18]; the low temperature stress consisted of an exposure for one day to 4 °C under a 16 h photoperiod (50 µmol·m^−2^·s^−1^·light). The second fully expanded leaf counted from the apex was sampled in each case. A wounding treatment was conducted by puncturing three leaves in five places with a size 10 (approximately 0.30 mm diameter) needle, and repeating this with ten further punctures after 24 h. The second true leaf was sampled 2 h after the second wounding event [38]. The phytohormone treatments involved spraying the leaves with either 20 µM ABA, 100 µM methyl JA or 10 µM SA. Control plants were sprayed with distilled water. Leaves were sampled at prior to the application of phytohormones, and then again after 1, 4, 8, 12 and 24 h. each time point stress/phytohormone treatment was imposed on three plants and each treatment was replicated three times. Control plants were kept at 22 °C. The sampled leaf material was snap-frozen in liquid nitrogen and stored at −70 °C.

### 3.2. Isolation and Sequencing of Full-Length CnAP2/ERF cDNAs

Total RNA was isolated using the RNAiso reagent (TaKaRa, Tokyo, Japan) following the manufacturer’s instructions. The first cDNA strand was synthesized from 1 µg total RNA using an M-MLV RTase cDNA Synthesis kit (TaKaRa) according to the manufacturer’s instructions. Primer pairs were designed (their sequences, along with all other PCR primer sequences used here are listed in Table 2) to amplify *CnAP2/ERF* fragments based on an EST sequence identified in a *C. nankingense* transcriptome database [39]. RACE PCR was then used to obtain the full-length cDNA. For the 3' reaction, the first cDNA strand was synthesized using the dT adaptor primer dT-AP, and this was followed by a nested PCR based on a specific primer pair and the adaptor primer AP. For the 5' reaction, the nested PCR used the primers AAP and AUAP provided with the 5' RACE System kit v2.0 (Invitrogen, Camarillo, CA, USA), along with gene-specific primers. For sequences, the amplicons were purified using an AxyPrep DNA Gel Extraction kit (Axygen, Shanghai, China) and cloned into the pMD19-T easy vector (TaKaRa). Finally, pairs of gene-specific primers were designed to amplify each open reading frame (ORF), which was also cloned into the pMD19-T easy vector for sequencing.

**Table 2:** PCR primer sequences utilized in this study.

Primer Name	Sequence (5' to 3')	Annotation
AP	AAGCAGTGGTATCAACGCAGAGTAC	Universal primers for 3' RACE
dT-AP	AAGCAGTGGTATCAACGCAGAGTACTTTTTTTTTTTTTTTT	
AUAP	GGCCACGCGTCGACTAGTAC	Universal primers for 5' RACE
AAP	GGCCACGCGTCGACTAGTACGGGIIGGGIIGGGIIG	
CnERF1-F	ATGCTTCTCTTCGGGGTCATTGCT	ORF of *CnAP2*/*ERF*s
CnERF1-R	TCACCAACTAGAACTACTGCTGCTGCT	
CnDREB1-1-F	ATGGCTTCAGCTACAATGGACTTAT	
CnDREB1-1-R	ATATACCCTCATAAACACTGCCACG	
CnDREB1-2-F	ATGGATGCACTAGAACCATTCATCAAGC	
CnDREB1-2-R	CTAGATAGAACCCCAATCAATCTCGTAC	
CnDREB1-3-F	ATGGAAGCACTTGAACCTTTTATCA	
CnDREB1-3-R	CTATAATGAACCCCAGTCAATCTCG	
CnDREB3-1-F	ATGAAATCCACAACATCCATCAGCG	
CnDREB3-1-R	TCACCAATAATTATTTCCATCCGGATC	
CnDREB3-2-F	ATGGACAGAAAATTAACAGACTGTCCATC	
CnDREB3-2-R	TCACCAATAATTTCCATCCGGATCTTCT	
EFIα-F	TTTTGGTATCTGGTCCTGGAG	qRT-PCR for *CnEFIα*
EFIα-R	CCATTCAAGCGACAGACTCA	
CnERF1-BamHI-F	CGGGATCCGGATGCTTCTCTTCGGGGTCATTG	Vector construction of *CnAP2*/*ERF*s
CnERF1-NOTI-R	TTGCGGCCGCGATCACCAACTAGAACTACTGCTGCTG	
CnDREB1-1-BamHI-F	CGGGATCCGGATGGCTTCAGCTACAATGGAC	
CnDREB1-1-NOTI-R	TTGCGGCCGCGAGATAGAACCCCAATCAATCTCGTA	
CnDREB1-2-BamHI-F	CGGGATCCGGATGGATGCACTAGAACCATTCAT	
CnDREB1-2-NOTI-R	TTGCGGCCGCGAGATAGAACCCCAATCAATCTCGTA	
CnDREB1-3-BamHI-F	CGGGATCCGGATGGAAGCACTTGAACCTTTTATC	
CnDREB1-3-NOTI-R	TTGCGGCCGCGATATTGAACCCCAGTCAATCTCGTAC	
CnDREB3-1-BamHI-F	CGGGATCCGGATGAAATCCACAACATCCATCAGC	
CnDREB3-1-NOTI-R	TTGCGGCCGCGATCACCAATAATTATTTCCATCCGG	
CnDREB3-2-BamHI-F	CGGGATCCGGATGGACAGAAAATTAACAGACTGTC	
CnDREB3-2-NOTI-R	TTGCGGCCGCGATCACCAATAATTTCCATCCGG	

### 3.3. Sequence Alignment and Phylogenetic Analysis

The presence of the AP2 domain in the isolated TFs was detected by a Blast search (available online: http://www.ncbi.nlm.nih.gov/index.html). *A. thaliana* *AP2/ERF* sequences were downloaded Arabidopsis thaliana transcription factor database [20], and combined with the newly acquired *CnAP2/ERF* sequences to perform a multiple alignment analysis based on ClustalW software [40]. The subsequent phylogenetic analysis relied on a Neighbor-Joining method, and a graphical representation was produced with the support of MEGA v5 software [41]. Internal branching support was estimated from 1000 bootstrap replicates.

### 3.4. Subcellular Localization of CnAP2/ERF

The plasmid for transient transformation was generated using the Invitrogen Gateway system, according to the manufacturer’s instructions. The *CnAP2/ERF* ORFs, lacking their stop codon, were amplified using a Phusion^®^ High-Fidelity PCR kit (New England Biolabs, MA, USA) (primers listed in Table 1), then cloned into the pMD19-T easy vector (Takara) and validated by DNA sequencing. Each of the isolated *CnAP2/ERF* TFs was inserted into the pENTR™ 1A vector (Invitrogen) using T4 DNA ligase (Fermentas, Burlington, ON, Canada), and the resulting construct recombined with pMDC43 to form the fusion vectors *CnERF1-GFP*, *CnDREB1*-*1*-*GFP*, *CnDREB1*-*2-GFP*, *CnDREB1*-*3*-*GFP*, *CnDREB3*-*1*-*GFP* and *CnDREB3*-*2*-*GFP* using the LR Clonase™ II enzyme mix (Invitrogen). Each plasmid DNA was then transiently introduced into onion epidermal cells, using a He-driven particle accelerator (PDS-1000; Bio-Rad, Hercules, CA, USA) according to the manufacturer’s instructions. As a control, an empty vector containing only the *GFP* sequence was transformed into similar epidermal cells. After bombardment, the onion peels were incubated for 16 h on Murashige and Skoog plates in the dark [42]. Green fluorescence was monitored by confocal laser microscopy [43].

### 3.5. Trans-Activation Activity Assay of CnAP2/ERF

The ORF (Open reading frame) of each *CnAP2/ERF* TF was amplified (primer sequences given in Table 1) and inserted into the *Not*I*/Bam*HI cloning site of the yeast expression vector pGBKT7 to produce *pBD-CnERF1*, *pBD-CnDREB1-1*, *pBD-CnDREB1-2*, pBD-CnDREB1-3, *pBD-CnDREB3-1* and *pBD-CnDREB3-2*. Either one of these constructs or an empty pGBKT7 (negative control) or pCL1 (positive control) plasmid was introduced into the yeast strain Y2Hgold (Clontech, Mountain View, CA, USA) [18]. Selection for transformants carrying either one of the *pBD-CnAP2/ERFs* or *pGBKT7* was carried out by culturing on SD/-Trp medium, while the *pCL1* transformants were selected on SD/-Leu medium. All three classes of transformant cells were transferred to an SD/-His-Ade medium supplemented with 20 mg/mL X-α-gal to observe cell growth. Since the expression of *His3* is regulated by the GAL4-BD region, a *CnAP2*/*ERF* TF possessing activation ability should bind to the GAL4 BD upstream promoter sequence of *His3*, thereby activating its expression and enabling the transformed cells to grow on SD/-His-Ade + 20 mg/mL X-α-gal medium [44].

### 3.6. Transcription Profiling of the CnAP2/ERF TFs

Transcription profiling was achieved using qRT-PCR, based on the CnAP2/ERF-RT-F/R primers listed in Table 1. The chosen reference gene was *EF1a* (Genbank accession number: KF305681). Each 20 µL reaction contained 10 µL SYBR^®^ Premix Ex Taq™ II (Takara), 0.2 µM of each primer and 10 ng cDNA template. The amplification regime consisted of an initial denaturation (95 °C/2 min), followed by 40 cycles of 95 °C/10 s, 55 °C/15 s, 72 °C/20 s [45]. A melting curve analysis was conducted following each assay to ensure the specificity of the amplicons. The qRT-PCRs were performed on three independent sets of RNA samples. Relative transcript abundances were calculated by the 2^−∆∆*C*t^ method [46]. The relative transcription levels of each *CnAP2/ERF* were log2 transformed, and the profiles compared using Cluster v3.0 software [47] and visualized using Treeview [48]. SPSS v17.0 software (SPSS Inc., Chicago, IL, USA) was used for all statistical analyses.

## 4. Conclusions

In summary, the six *C. nankingense* AP2/ERF TFs appear to have distinct roles as they responded differentially to the various stresses and hormonal treatments. Our results lay the basis for further investigation into the regulatory mechanism of CnAP2/ERF genes in various stresses.

## Supplementary Materials

Supplementary materials can be found at http://www.mdpi.com/1422-0067/16/01/2052/s1.
